# Participant-reported priorities and preferences for developing a home-based physical activity telemonitoring program for persons with tetraplegia: a qualitative analysis

**DOI:** 10.1038/s41394-019-0188-6

**Published:** 2019-05-16

**Authors:** Renee Pekmezaris, Andrzej Kozikowski, Briana Pascarelli, John P. Handrakis, Ashley Chory, Doug Griffin, Ona Bloom

**Affiliations:** 1 Division of Health Services Research, Center for Health Innovations and Outcomes Research, Department of Medicine, Donald and Barbara Zucker School of Medicine at Hofstra/Northwell, Manhasset, NY USA; 2 The Feinstein Institute for Medical Research, Manhasset, NY USA; 3 Department of Physical Therapy, New York Institute of Technology, Old Westbury, NY USA; 4 VA RR&D National Center for the Medical Consequences of Spinal Cord Injury, James J. Peters VA Medical Center, Bronx, NY USA; 5 Sports Therapy and Rehabilitation Services (STARS), Northwell Health, East Meadow, NY USA; 6 Department of Physical Medicine and Rehabilitation, Donald and Barbara Zucker School of Medicine at Hofstra/Northwell, Manhasset, NY USA; 7 Present Address: National Commission on Certification of Physician Assistants, 12000 Findley Road, Suite 100, Johns Creek, GA 30097 USA

**Keywords:** Outcomes research, Translational research

## Abstract

**Study design:**

Focus group.

**Objectives:**

The purpose of this qualitative study was to explore perceptions and priorities of persons with spinal cord injury (SCI) for physical activity and to incorporate their feedback to inform future development of a physical activity program delivered via a telemonitoring platform.

**Setting:**

New York.

**Methods:**

Qualitative data were collected from a purposive sample of adults with tetraplegia (*N* = 7). Two investigators led an audio-recorded focus group using a moderator’s guide. Data were analyzed using a six-phase thematic analysis approach.

**Results:**

The discussion focused on two major areas, which resulted in multiple derived themes and subthemes. The first theme centered on the daily life of persons with tetraplegia, including changes after SCI, gain of function prioritization, and identification of psychosocial support systems that facilitate community reintegration after injury. The second theme centered on participant perceptions and recommendations for a physical activity program delivered via a telemonitoring platform. Desired design features included variations in schedule, diverse activities, or exercises included in each class, and optional two-way video to enable social interactions with classmates.

**Conclusions:**

Participants favorably viewed the concept of a physical activity program delivered via a telemonitoring platform and contributed program design ideas. Although this was a small sample size, challenges to obtaining physical activity expressed by participants were consistent with those identified previously in larger studies of persons with tetraplegia. Therefore, we expect these concepts and their recommendations to be relevant to the greater SCI community.

## Introduction

Approximately 350,000 persons in the US are living with traumatic spinal cord injury (SCI) [1, 2]. Due to reduced mobility, persons with SCI are at increased risk for developing obesity, muscle atrophy, osteoporosis, accelerated atherogenesis, type II diabetes mellitus, and other medical consequences that increase the risk of stroke and coronary heart disease [1–3]. This reduced mobility often has deleterious psychosocial effects that impact quality of life, including increased social isolation, reduced social participation, reduced exercise self-efficacy, and depression [4, 5]. Thus, there is a critical need for therapeutic strategies that reduce the risk of multiple medical and psychosocial consequences of SCI.

Physical activity is a recommended therapeutic strategy to reduce risks of common medical consequences across diverse clinical populations [6, 7]. Physical activity reduces risks of coronary heart disease and diabetes, increases immunity and blood circulation, and decreases inflammation, fat, anxiety, pain, and improves mood and sleep [8–11]. The American College of Sports Medicine recommends that able-bodied adults perform 150 min of moderate-intensity aerobic exercise and participate in two or more days of muscle-strengthening exercise weekly [12]. The latest physical activity guidelines for adults with SCI recommend, “at least 20 min of moderate to vigorous-intensity aerobic exercise two times per week and three sets of strength exercise for each functioning muscle group, at moderate to vigorous intensity, two times per week” [13, 14]. For cardiometabolic health benefits, it is recommended that adults with SCI engage in at least 30 min of moderate to vigorous-intensity aerobic exercise three times per week [13, 15].

Persons with SCI and other disabilities are less likely to engage in regular physical activity, due to many modifiable barriers. These include: lack of knowledge about existing programs/safe exercises, insufficient programming, lack of transportation, cost, and scheduling issues [16]. There are also other barriers, such as feeling too hot or cold outdoors or distance from an adaptive sports facility [17].

In the general population, telemonitoring approaches to delivering physical activity are part of a highly successful commercial fitness industry. Consumers are offered the ability to choose a program to engage in at home, with recorded or live classes, that can be delivered to a TV, tablet, phone, or computer via a commercial internet provider. Compared to a gym membership, telemonitoring is convenient, scalable, and relatively low cost. Regardless of the modality, telemonitoring physical activity programs often require minimal exercise equipment and are delivered at home on a personalized schedule.

In addition to the physical health benefits, such as increased muscle strength and improved cardiovascular fitness, many physical activity instructors also engage actively in motivational strategies, to promote adherence and increase exercise self-efficacy [18]. Increasingly, telemonitoring enables a participant to experience self-efficacy in the following ways: (1) Mastery of experiences, the strongest predictor of self-efficacy, relate to actual performance when successfully meeting a challenging task. Participants performing daily health behaviors and seeing progress, experience mastery. (2) Vicarious modeling (seeing others facing similar challenges and reaching their goals) will be achieved by viewing other participants of similar abilities attaining activity goals. (3) Social persuasion (verbal encouragement) is provided by the instructor. (4) Physiological factors, such as anxiety and distress, can be experienced by participants when they fail to meet activity goals; the instructor can interpret this as situational and not associated with overall success [19, 20].

Home-based physical activity delivered via telemonitoring may be a particularly useful option for persons with SCI as a way to modify common environmental barriers to achieve the benefits of regular physical activity [21]. To address these and other barriers, telehealth approaches are being increasingly studied in the context of SCI [21]. Sweet and colleagues are starting an RCT of an 8-week tele-rehab program for persons with paraplegia to measure changes in psychosocial variables related to exercise participation and quality of life [22]. Another study measured the effects of a home-based exercise program in persons with chronic SCI, including outcome measures of metabolism, body composition, physical activity, energy intake, measures of health and wellbeing, resting metabolic rate, heart rate, and blood pressure, aerobic capacity, immune function, and adipose gene expression [23]. Encouraging results using telemonitoring have been obtained across physical health measures (i.e., wound care), as well as psychological health [21].

There is a need to establish novel methods to facilitate regular physical activity for persons with SCI [24]. Here, we report the results of a qualitative study of priorities and preferences for developing a home-based physical activity telemonitoring program for persons with tetraplegia. We consider this to be a first step towards optimizing feasibility and acceptability in a physical activity program for persons with SCI [13].

## Methods

### Design

This is a qualitative study of adults with chronic (at least 1 year from injury) tetraplegia who were recruited from the NY metropolitan area. The rationale for including only persons with tetraplegia was because, in general, this group has fewer opportunities for achieving physical activity in their daily life, lower reference values of cardiovascular fitness (relative VO2 peak), higher risk factors for cardiovascular disease, and lower life expectancy than persons with paraplegia.

A short demonstration video developed by the study team was presented to participants to show the concept of a telemonitoring physical activity program led by a physical therapist for persons with tetraplegia. Moderators explained that they envisioned that participants would join the class via a tablet with a split screen that showed themselves, the instructor, and classmates conducting exercises. Moderators described that an instructor would monitor vital sign data (heart rate and blood oxygenation) of participants in real time via a pulse oximeter. Before engaging in exercise, participants would be trained on proper equipment use. For safety, participants would be asked by the instructor every 5 min during the intervention, to describe any symptoms of discomfort, including pain (musculoskeletal or other), fatigue, shortness of breath, or dizziness.

Frequency, duration, and type of proposed activities are based on the most recent guidelines on physical activity for persons with SCI [24]. The intervention presented was proposed to be delivered three times/week for 45 min, with ≥30 min of activity. The circuit training program proposed was based on evidence of strength and cardiorespiratory benefit in persons with SCI [25]. Stretching, cardiovascular, and strengthening exercises would be tailored to participants’ functional abilities. TheraBand, with open handgrips (loops), would be used to provide resistance for strength training [26]. Moderators explained that the program would consist of three repetitions of: (A) Warm-up: a series of active (nonresisted) movements: shoulder lateral raises, flies, shoulder rolls, wide biceps curls, shoulder shrugs, triceps extensions to rear; (B) Circuit exercise program: resistance followed by aerobic (arm spinning) exercises with rest periods as needed (~15 s). Resisted movements would include: Set 1: seated rows, horizontal shoulder abduction, arm spinning/circles (aerobic exercise), Set 2: shoulder internal rotation, shoulder external rotation, aerobic exercise, Set 3: straight arm pulldowns, chest press, aerobic exercise [26].

### Data collection

A 3-h focus group was conducted in January 2018, led by two moderators previously unknown to participants. Moderators used a moderator guide with open-ended questions and probes, related to a range of relevant topics including experiences with and priorities for benefits of physical activity before and after their injuries, technology use, and perceptions of important features that should be incorporated into a telemonitored physical activity program. The discussion was digitally recorded (using two recorders in case of technical failure), stored on an internal password protected server to ensure security, and transcribed professionally. Transcripts were checked against the original recordings for accuracy.

### Data analysis

To optimize credibility, transferability, and dependability of results, we utilized analyst triangulation, peer debriefing, and conducted an audit trail of decisions made during the analysis and rationale. The transcript was analyzed by two researchers (A K and B P), to achieve triangulation to gain a more complex understanding of the data. A six-phase thematic analysis approach was utilized [27, 28]. In the first phase, transcripts were reviewed independently multiple times to become familiar with the data. Researchers documented initial theoretical and reflective thoughts, and potential codes and themes. In the second phase, researchers focused on data patterns and generated a comprehensive set of codes through inductive and deductive coding. Two researchers documented their reasoning for coding blocks of text from the transcript of the focus group to explain how the data were perceived and examined. The third phase consisted of searching for themes after coding and codes were collated. In the fourth phase, themes were reviewed and refined. Criteria for retaining themes were that they needed to be specific enough to be concrete, while broad enough to capture ideas. Themes with sparse data were eliminated and those with large amounts of data were further divided into separate themes. In the fifth phase, team members met and discussed the finalization of theme names. In the sixth phase, the report was generated.

## Results

Participants were persons with tetraplegia (*N* = 7: 5 males and 2 females) who were wheelchair users for community mobility. The discussion explored challenges of living with tetraplegia, gain of function prioritization, social networks, and design recommendations for a telemonitored physical activity program.

Participants were asked to rank their gain of function prioritization on a seven-point scale, with one being most, and seven being least important, in the following areas: arm/hand function, upper body/trunk strength and balance, bladder/bowel function, lived experiences of sexual function, elimination of chronic pain, sensation and mobility (“mobility could be anything that gets your body around in space”) [29]. Most participants ranked either arm/hand function, sensation, or improvement of mobility as the most important. The next gain of function priorities ranked was upper body/trunk strength and balance, elimination of chronic pain, and sexual function. Two major discussion themes emerged from a six-phase thematic analysis approach to the transcript.

### Theme one: daily challenges

#### Pain

Several participants described challenges of performing activities of daily living (ADLs) while experiencing constant pain. The locations of pain symptoms varied by individual, including the back, neck, shoulders, and feet. Multiple participants reported that pain symptoms were worse in the morning and did not resolve completely throughout the day.

*“Right now, I feel like someone’s kicking me in the back but that’s normal for me, so it’s just one of those things you kind of deal with…”*

*“…I have chronic back pain that just will not go away. It’s probably – if I say on a scale of one to ten, it’s probably around a good eight most of the day…”*

*“I’m in pain every day when I get home. I’m in bed by 7:00 because I can’t even function.”*

#### Fatigue

Participants also discussed how pain impacted feelings of fatigue and strategies to cope with interruptions in sleep.

*“I can’t sleep on my back or on my stomach, so I’m like on one side until – oh, that’s burning. Then if I switch to the other side until that shoulder is burning and I sleep the worst. I’m up every hour and a half. … I don’t sleep. I nap during the day a lot, quite frankly… because that’s the only time I can actually sleep quite honestly is in my car with my car [seat] back listening to the radio while I’m between picking up or dropping kids back or errands, that kind of thing.”*

*“And you talk about getting exhausted during the day. I want to sleep every day by 12:00. But I’m at work, so I can go in my office for a little while just to try to rest for a minute.”*

#### Changes in physical activity before and after injury

Multiple participants reported being athletic and active prior to their SCI, had careers in physically enduring professions or participation in active sports including swimming, motocross, running, cycling, and skiing.

*“Yeah, motor cross. Yeah. So I used to ride-a lot of cycling, a lot of swimming. I had a home gym that I worked out in all the time. Running-I was a terrible runner because my knees weren’t that great. So I would run a little bit, but not that much. Mostly cycling. I loved cycling. Anything with wheels, I was there.”*

*“I was very free spirited, I’d say. We’ll put it that way. But yeah, I sort of was very spontaneous and enjoyed flying by the seat of my pants and all that. It’s like losing a little piece of you.”*

As expected, the intensity and type of physical activity changed for most participants after injury. Most focus group participants were not engaged in regular physical activity, outside of the exercises prescribed during physical therapy. For participants who were active, post-injury activities include using a stationary bike, Thera-bands for resistance training, and free weights.

*“When I first came home, I was doing them every day. And then little by little, you’re slacking off. But like I said, every day, once I get into bed, that’s when I do the most Thera-bands or weights or anything because I’ll put a wrist [adaptor] on my arm. I’ll go on my side and I’ll do the left arm. Then I get turned the other way, I’ll do the right arm.”*

*“...Everything from Thera-bands like you were talking, to cuff weights. I use cuff weights as well that-most people use them on their ankles when they’re running or exercising, but they also work great for quads around your arm. It’s like a Velcro weight. The Rickshaw [wheelchair rehab exercise machine]…. It’s a great machine for people in wheelchairs. And they have another machine there which is called the upper tone…It’s kind of a home gym-type looking machine that’s specifically designed for people in wheelchairs and people with limited hand function.”*

#### Physical therapy

Variation in physical therapy and interactions with physical therapy personnel were discussed and perceived as impacting the post-injury rehabilitation process.

*“I’ve been to great therapists and I’ve been to not-so-great therapists, and what they did clinically was not that different from each other. The difference was the therapists’ behavior, the interaction.”*

*“I mean, it felt like it’s a total package there. You get a lot of focused attention. You – and they – start on the dime and they give you every second of that hour.”*

#### Social support and emotional health

Participants discussed the critical role of social networks (family and friends) in community reintegration after injury. In addition, participants were motivated and inspired by interacting with peers with SCI who demonstrated resilience.

*“…when you see people getting better, it helps. It makes you believe you can do the same thing too.”*

The importance of self-efficacy to obtaining functional gains was also discussed, including the importance of maintaining both physical and emotional health. The feelings of well-being obtained from exercise were reported to reinforce the desire to continue exercising.

*“When I’m in a good mood, I feel I can conquer the world. But when I’m in a lousy mood – like today is not a great mood for me – is I don’t feel good about anything, and I don’t want to do anything because I’m miserable. But then tomorrow I’ll feel great and say I can pretty much take on the world and do anything I want and just let me do what I got to do.”*

*“Yeah, inspirational. Yeah, it would raise inspiration, want me to build more muscle on my end to want to feel better and know that I’m healthier and to keep going for whatever reason, whether it’s for walking or not.”*

*“And I can tell you personally that I should probably be further along physically than I am… I think I plateaued and then went the other direction because of my own inability to push those things out of my mind…like if your head’s not there, like in anything in life, but especially with SCI rehab, it’s hard enough knowing that this happened.”*

#### Individualized goals and gain of function prioritization

Participants discussed the importance of recognizing that goals and priorities may vary by individual and that each person will begin the program looking for a different outcome. For some, success may be defined as an improvement in mobility, whereas for others success may be defined as increased social interactions, motivation, or health maintenance.

*“I think everybody’s priorities and everybody’s goals are different.”*

*“So I mean, so I don’t want to lose those [functions] any worse than they’ve been getting over the years because it’s like almost limited to what I can kind of do…”*

*“Well, I guess it depends on somebody’s lifestyle and age has a lot to do with it. So I would say some people are just looking to maintain themselves and stay healthy to be able to continue to do the activities that they currently do.”*

*“Yeah, and just feeling better as well in daily activities.”*

### Theme two: design recommendations

#### Interaction with other classmates

A strong recommendation was made to foster potential interactions among classmates in order to motivate and inspire one another. An additional recommendation was to include a feature to extend class times to allow for social interactions among classmates before or after exercise. Some participants suggested that two-way viewing among classmates should be optional, so as to not discourage those who might feel uncomfortable.

*“I think it would be key to interact with not only the therapist, but with other patients. So I see Jack on the one screen and he’s struggling, I’m like come on. Do one more. Do one more. And we’re all telling him—me, Alex—Jack, come on. Do one more. And he pulls through, so it gives that mental back.”*

*“So you said a 45-minute designed program, but maybe its 60* *min and we all log on 15* min before. We could all- oh, Maria*, how’s that going, or Chris*, how’s that? So the social muscle to it instead of just working on arms and then logging off, like good old talk.”

*“But to have the ability to [see others] should certainly be an option… But they should at least have the option to turn it off if they want I think, right?”*

#### Equipment training and orientation

Participants suggested that it would be helpful for someone to orient a class member, assist with equipment needs and demonstrate specific exercises included in the program prior to session initiation.

#### Individualized adaptations

Participants suggested that including a variety of exercises within each class would be desirable, in order to meet personal preferences and to address varying physical abilities. Multiple participants suggested that three times per week would be the preferred frequency of classes, held at a variety of days and times to accommodate different schedules (e.g., weekday, weekend, and evening sessions).

## Discussion

The goal of this focus group was to discuss experiences with physical activity and gather input from persons with tetraplegia to inform future design of a physical activity program delivered via telemonitoring that would be feasible, acceptable, and consistent with exercise guidelines for those with SCI. A minor aspect of the discussion revealed that, in general, priorities for improvement included: arm/hand function, sensation, and improvement of mobility as being most important. In addition, upper body/trunk strength and balance, elimination of chronic pain, and improving lived experiences of sexual function were also ranked as important. Data demonstrate that different modalities of exercise and physical activity have indeed been shown to improve aspects of physical capacity, health, and abilities to perform activities of daily living (e.g., functional wheelchair maneuvers and transfers) in persons with SCI [30].

Participants perceived a home-based physical activity program as needed and important. Intensity and type of physical activity performed before and after injury were discussed. Participants identified family support, psychological state, and having a peer network (e.g., others with SCI) as important factors for their overall recovery. Participants regarded their input and feedback as critical for ensuring usability and feasibility, including the ability to make choices regarding whether a participant can be seen by classmates, types of exercises in a class, and timing of class delivery to suit multiple schedules. Participants generally expressed enthusiasm for interacting with classmates, a desire for help from a caregiver or professional in initial set up, and comfort with a frequency of three times/week for classes and a duration of 45–60 min per class.

## Conclusions

Participants perceived multiple potential benefits of a physical activity for persons with SCI delivered via telemonitoring. Participants had several practical suggestions to optimize design and delivery of such a program. Clearly, a pilot study in this population testing this kind of intervention is needed. It is important that future studies incorporate feedback from participants on the design and implementation of a physical activity program.

### Data archiving

Data generated during the focus group are not publicly available in order to protect privacy of participants. De-identified data can be made available upon request to the corresponding author.
